# Optimization of DNA extraction and PCR protocols for phylogenetic analysis in *Schinopsis* spp. and related Anacardiaceae

**DOI:** 10.1186/s40064-016-2118-4

**Published:** 2016-04-18

**Authors:** Virginia Y. Mogni, Mariano A. Kahan, Luciano Paganucci de Queiroz, José L. Vesprini, Juan Pablo A. Ortiz, Darién E. Prado

**Affiliations:** Facultad de Ciencias Agrarias, Universidad Nacional de Rosario, Campo Experimental Villarino, S2125ZAA Zavalla, Santa Fe Argentina; IICAR, Consejo Nacional de Investigaciones Científicas y Técnicas (CONICET-UNR), Zavalla, Argentina; SINERGIUM Biotech, Ruta Panamericana Km 38.7, 1619 Garin, Buenos Aires Argentina; Departamento de Ciências Biológicas, Universidade Estadual de Feira de Santana, Av. Transnordestina, s/n, Novo Horizonte, Feira de Santana, Bahia 44036-900 Brazil

**Keywords:** Anacardiaceae, DNA isolation, Phylogeny, *Schinopsis*, Sequencing

## Abstract

The Anacardiaceae is an important and worldwide distributed family of ecological and socio-economic relevance. Notwithstanding that, molecular studies in this family are scarce and problematic because of the particularly high concentration of secondary metabolites—i.e. tannins and oleoresins—that are present in almost all tissues of the many members of the group, which complicate the purification and amplification of the DNA. The objective of this work was to improve an available DNA isolation method for *Schinopsis* spp. and other related Anacardiaceae, as well as the PCR protocols for DNA amplification of the chloroplast *trn*L-F, *rps*16 and *ndh*F and nuclear ITS–ETS fragments. The modifications proposed allowed the extraction of 70–120 µg of non-degraded genomic DNA per gram of dry tissue that resulted useful for PCR amplification. PCR reactions produced the expected fragments that could be directly sequenced. Sequence analyses of amplicons showed similarity with the corresponding *Schinopsis* accessions available at GenBank. The methodology presented here can be routinely applied for molecular studies of the group aimed to clarify not only aspects on the molecular biology but also the taxonomy and phylogeny of this fascinating group of vascular plants.

**Electronic supplementary material:**

The online version of this article (doi:10.1186/s40064-016-2118-4) contains supplementary material, which is available to authorized users.

## Background

The Anacardiaceae is a cosmopolitan family including 81 genera and approximately 800 species (Pell et al. 2011). Many genera are important for having edible fruits (*Anacardium*, *Mangifera*, *Pistacia*, *Sclerocarya*, *Spondias*), ornamental use (*Cotinus*, *Rhus*, *Schinus*), or quality timber (*Astronium*, *Schinopsis*). Some genera have resiniferous channels accumulating tannins, phenolic compounds and oils that increase the wood resistance.

The South American small genus *Schinopsis* Engl. is economically important given its extremely tough and durable timber. Its species have ecological relevance since they are usually forest dominants (Barberis et al. 2012). Although a new species was recently described (Mogni et al. 2014), the taxonomy and phylogeny of *Schinopsis* is not well resolved. Classical studies based on morphology have limitations due to low variation between species and the existence of interspecific hybrids (Mogni 2015). Therefore, integrating morphological with molecular approaches could help resolve this issue.

Molecular sequence data have revolutionized phylogenetic analysis. In vascular plants, most sequenced-based molecular phylogenetic studies rely on DNA regions of the plastid genome, and on internal (or external) transcribed spacers (ITS/ETS) regions of the 18S–5.8S–26S nuclear ribosomal cistron. The chloroplast *trn*L-F, *ndh*F and *rps*16 regions and nuclear ITS and ETS have been used in Anacardiaceae (Pell 2004; Nie et al. 2009; Xie et al. 2014; Weeks et al. 2014; Machado et al. 2015) and related families such as Burseraceae (Becerra and Venable 1999; Weeks et al. 2014) and Meliaceae (Koenen et al. 2015). Nevertheless, few *Schinopsis* accessions have been included in those studies, due to the presence of tannins and oleoresins that strongly affect DNA purification, PCR amplification and sequencing, as it happens in other Anacardiaceae (Pell pers. com.) or other plant groups (Permingeat et al. 1998). Such is the *Spondias* case, reported to be exceedingly difficult to purify and amplify DNA even from fresh leaf samples (Mitchell and Daly 2015).

Several methods for DNA extraction of plants with high phenolic contents were developed (e.g. Porebski et al. 1997; Permingeat et al. 1998). Nevertheless, in *Schinopsis* spp. attempts carried out using this kind of protocols were unsuccessful (Kahan 2007). The lack of specific methodology for these species has lead to uncertain results and consequently delayed the application of molecular analyses involving numerous accessions and/or species. Moreover, due to the wide geographic distribution of these species, the utilization of herbarium specimens or silica gel-dried material is mandatory.

The aim of this work was to develop an adapted protocol for routine isolation of DNA and to optimize a PCR protocol for amplifying chloroplast and nuclear regions useful for molecular phylogenetic analysis in *Schinopsis* spp. and other Anacardiaceae.

## Results and discussion

**Fig. 1:**
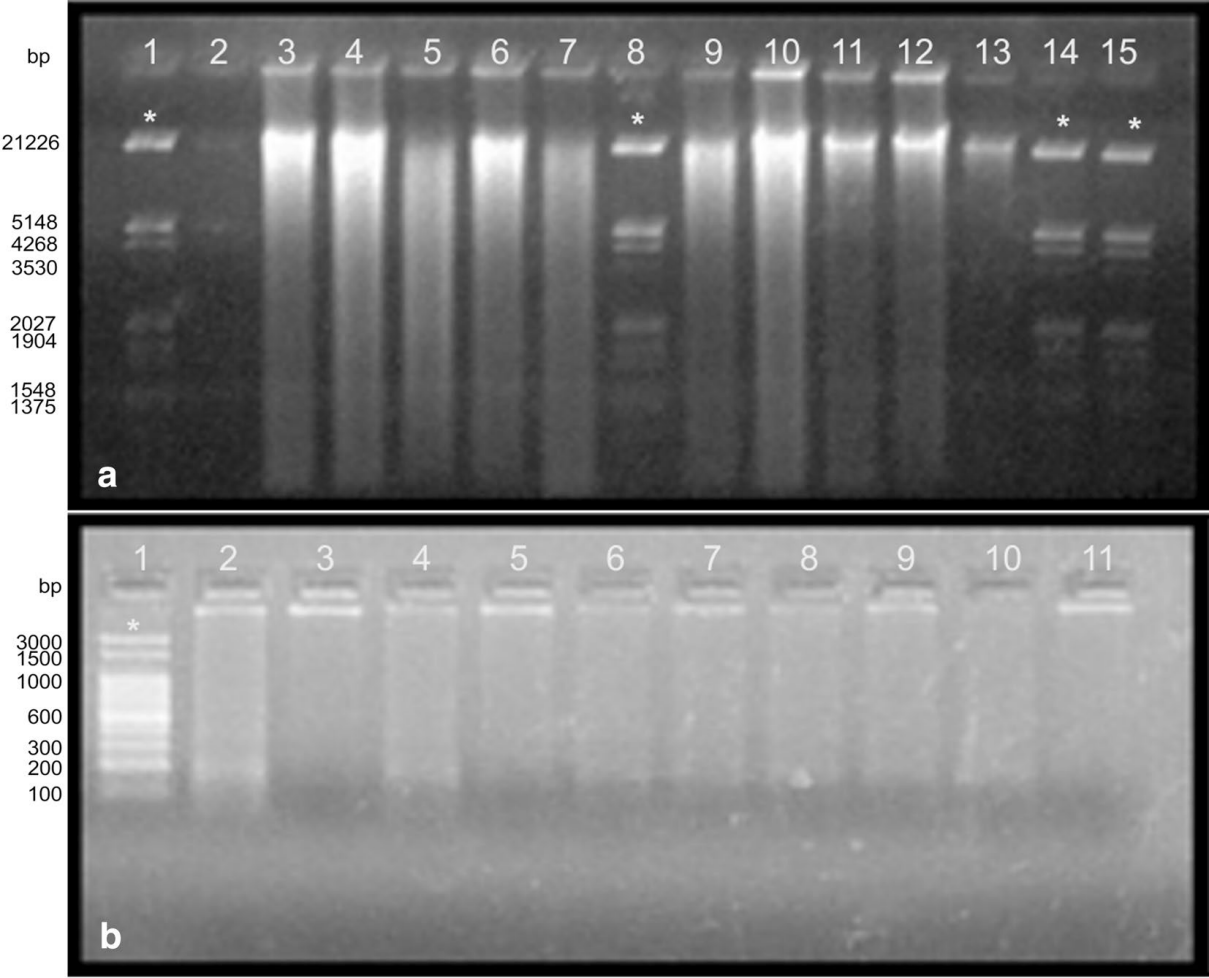
0.8 % Agarose gel electrophoresis depicting results of DNA extraction of some accessions of *Schinopsis* spp. and related species. **a** DNA extracted with the optimized protocol. *Lanes* *1*, *2*, *8*, *14* and *15* molecular marker (Lambda *Eco*RI/*Hin*dIII), *3* and *4* *S. lorentzii*, *5* and *6* *S. marginata*, *7* *Lithraea molleoides*, *9*–*13* *S. brasiliensis*. **b** DNA extracted with the DNAEasy Plant Mini Kit (Quiagen Inc. Valencia, CA) used as control. *1* Molecular weight marker (100 bp DNA Ladder), *2*–*11* different specimens of *S. brasiliensis* (*asterisk* reference bands of the marker)

Briefly, the modifications introduced to the protocol described by Permingeat et al. (1998), that allowed the isolation of total DNA of all species tested were the following: the decrease of the initial quantity of plant material (20–25 vs. 500–1000 mg); the addition of sterile sand (or liquid nitrogen in the Eppendorf tubes) for disrupting leaf tissue and create the lysate; the extension in the incubation time and the temperature increment (150 min at 75 °C vs. 60 min at 60 °C) of the Extraction Buffer; the duplication of the chloroform step for protein removal and the final precipitation with Ethanol in presence of 5 % V/V NaCl 5 M (instead of NaAc 3 M pH 5.2). The result of the extraction methods are shown in Fig. 1. The modified protocol produced clear bands of high molecular weight corresponding to the total DNA in most of the accessions (38/41), although some samples showed smearing consistent with partially degraded DNA (Fig. 1a). The assays performed with the DNeasy Plant Mini Kit (control) showed similar results and allowed the extraction of the 12 samples tested as well, and less smearing was observed (Fig. 1b). Comparison by eye of ethidium bromide fluorescence produced by the samples to the Lambda DNA and spectrophotometric quantification showed values ranging from 70 to 120 µg of DNA per gram of tissue (vs. 75–130 µg obtained with the control, see Fig. 1b), indicating a relative good yield and the presence of high molecular weight DNA, and that the samples can be used directly for PCR reactions.

**Fig. 2:**
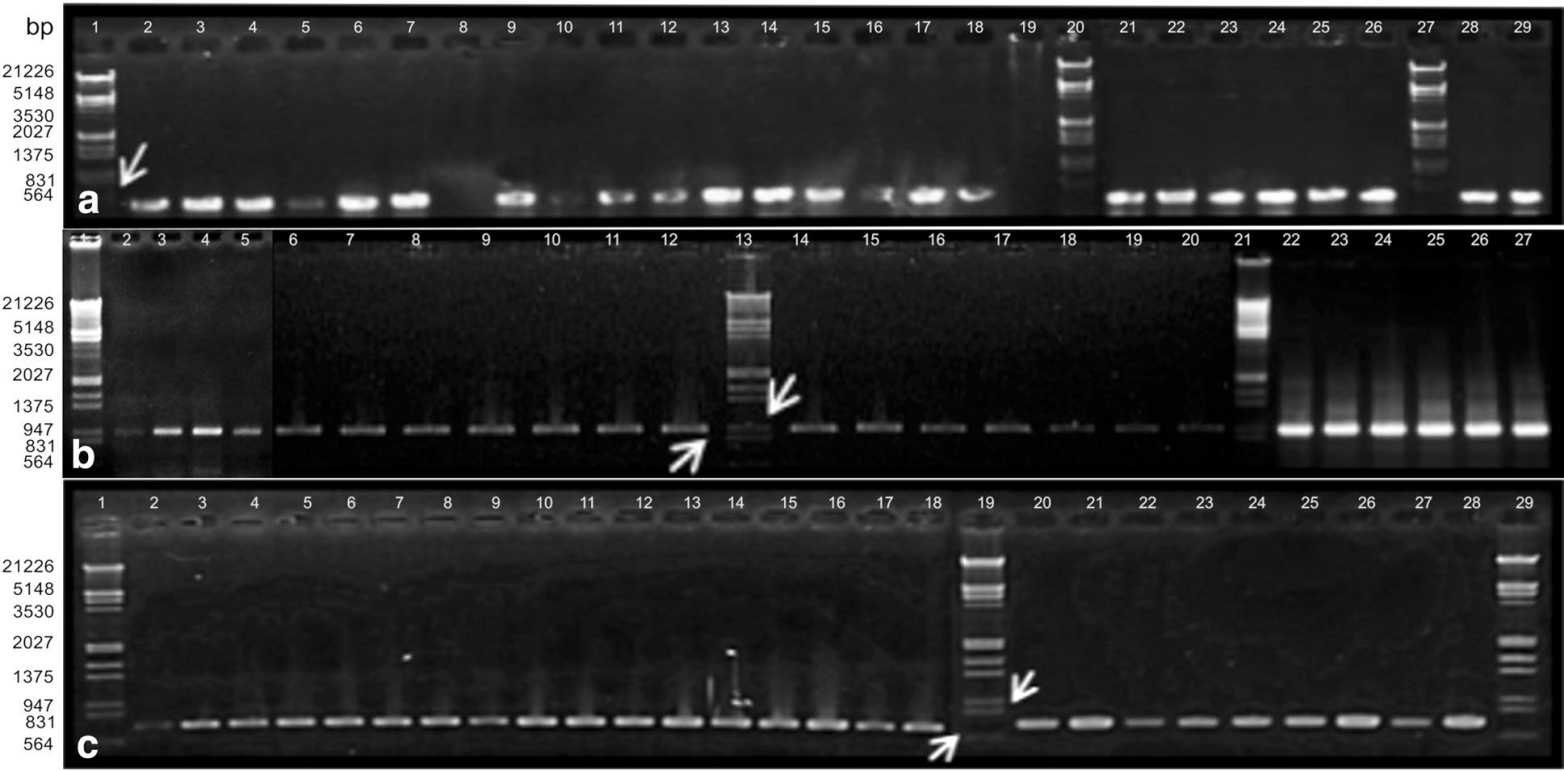
1.5 % Agarose gel electrophoresis of PCR products of several accessions of *Schinopsis* spp. and related species. **a** *trn*L-F amplified in two parts. *Lanes* *1*, *20*, *27* molecular marker, *2*–*5* *S. boqueronensis*, *6*–*7*, *9* *S. cornuta*, *10* *S. peruviana*, *11*–*14* *S. heterophylla*, *15*–*18*, *21* *S. brasiliensis*, *22*–*23* *S. marginata*, *24* *S. lorentzii*, *25*–*26* *S. brasiliensis*, *28*–*29* *Astronium urundeuva* and *Apterokarpos gardneri*, and *8*, *19* without sample. **b** *rps*16. *Lanes* *1*, *13* and *21* molecular marker, *2* S. *peruviana*, *3*–*6* *S. heterophylla*, *5*–*9*, *14*–*15* *S. brasiliensis*, *10*–*11* *S. marginata*, *12* *S. lorentzii*, *16*–*17* *Astronium urundeuva* and *Apterokarpos gardneri*, *18*–*19* *S. balansae*, *20* *S. lorentzii*, *22*–*24* *S. boqueronensis*, *25*–*27* *S. cornuta*. **c** *ndh*F. *Lanes* *1*, *19* and *29* molecular marker, *11*, *12*, *17*–*19* *Astronium urundeuva*, *Apterokarpos gardneri*, *Lithraea molleoides*, *Loxopterygium grisebachii* and *Schinus areira*; other lanes, *Schinopsis* spp. *Arrows* indicate the reference bands of the marker Lambda *Eco*RI/*Hin*dIII (564 bp in **a**, 947 and 831 in **b** and 831 and 564 in **c**) to estimate the amplicons size

**Fig. 3:**
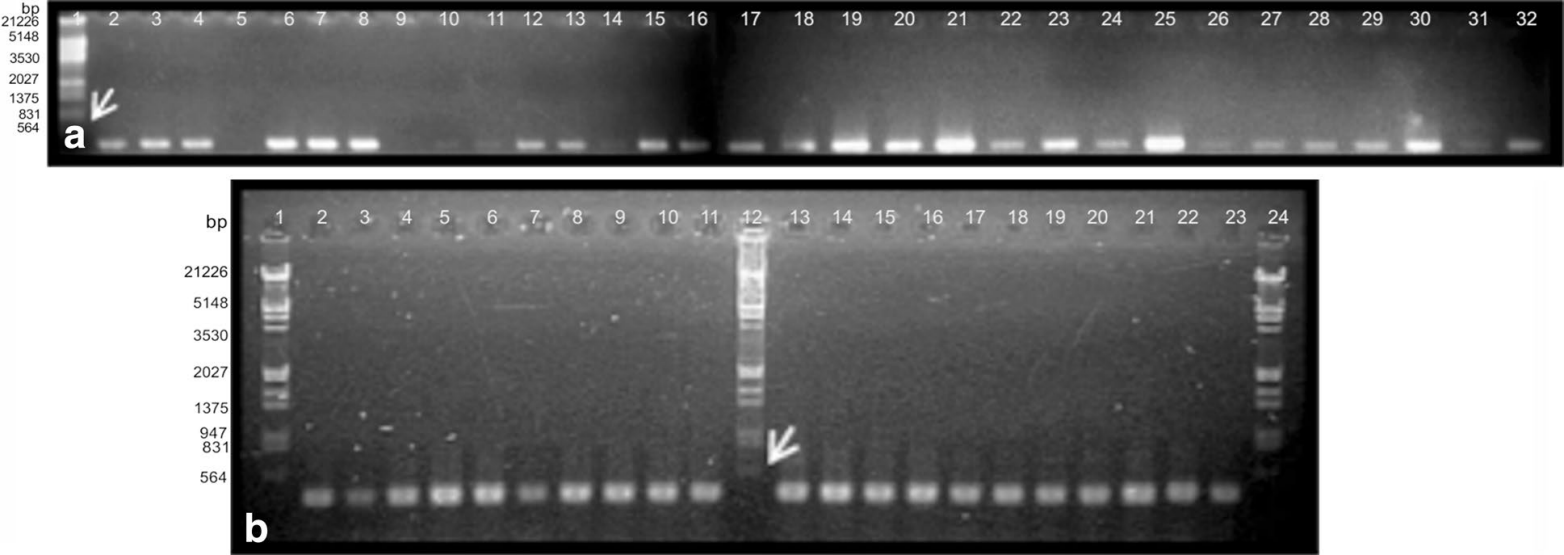
1.5 % agarose gel electrophoresis of PCR products of some accessions of *Schinopsis* spp. and related species. **a** ETS. *Lane 1* molecular marker, *2*–*5* *S. boqueronensis*, *6*–*8*, *S. cornuta*, *9*, S. *peruviana*, *10*–*13*, *S. heterophylla*, *14*–*18*, *21*, *22*, *S. brasiliensis*, *19*, *20*, *S. marginata*, *21*, *27*, *28* *S. lorentzii*, *23*, *24*, *29*–*31* *Astronium urundeuva*, *Apterokarpos gardneri*, *Lithraea molleoides*, *Loxopterygium grisebachii* and *Schinus areira*. **b** ITS2. *Line 1*, *12*, *24* molecular marker, *2* *S. cornuta*, *3*–*6* *S. heterophylla*, *7*–*10*, *15*, *16* S. *brasiliensis*, *11*, *12* *S. heterophylla*, *13*, *18*, *19*, *S. lorentzii*, *20*–*22*, *Lithraea molleoides*, *Loxopterygium grisebachii* and *Schinus areira*. *Arrows* indicate the reference bands of the marker Lambda *Eco*RI/*Hin*dIII (564 bp in **a** and **b**) to estimate the amplicons size

PCR assays performed using the DNA preparations diluted 1/10 allowed the generation of all fragments tested. The addition of BSA 1/1000; MgCl_2_ 5 mM and DMSO 1 M (for ETS and ITS) in the PCR mixture was crucial for amplification success, as it was previously reported (Savolainen et al. 1995; Baldwin et al. 1995; Särkinen et al. 2012). This is probably because BSA has a high content of lysine; it joins phenolic compounds when added to the PCR mix, avoiding *Taq* polymerase inactivation (Kreader 1996). On the other hand, DMSO acts by relaxing the typical secondary structure of nuclear ribosomal regions during amplification (Álvarez and Wendel 2003). Chloroplast regions *trn*L-F, *rps*16 and *ndh*F resulted in amplicons of 400, 900 and 650 bp respectively (Fig. 2a–c). On the other hand, the amplification of the nuclear region ETS resulted in a fragment of approximately 300 bp (Fig. 3a), and ITS2 in 200–300 bp (Fig. 3b).

Amplification products corresponding to each fragment from the different accessions were directly sequenced. Most sequences showed similarity with *Schinopsis* accessions available at GenBank, particularly to those obtained by Pell (2004). Chloroplast regions displayed high scores, with E-values and identities of 0.0 and 98 % respectively for *trn*L-F; 0.0 and 99 % for *rps*16 and 0.0 and 99 % for *ndh*F. Likewise, nucleic sequences showed E-values and identities of 1E^−129^—99 % for ETS and 2E^−94^—90 % for ITS2. Consequently, the amplicons corresponded to both expected chloroplast and nuclear hypervariable regions of *Schinopsis*.

## Conclusions

Based on Permingeat et al. (1998), we developed an adapted new protocol to isolate DNA from dried *Schinopsis* leaves. Our experiment revealed that the modifications introduced (see *Protocol*, “Methods” section) were favourable to improve the DNA isolation. Although some degradation was observed, sufficient quantity of high molecular weight DNA was available in most samples. Moreover, the quality of the DNA isolated was sufficient for PCR amplification. Interestingly, PCR products could be sequenced directly, without necessity of isolation, purification and cloning. Most sequences matched with the corresponding subject in the data bank, thus indicating its specificity.

The results presented in this work have an interesting potential use for molecular studies of Anacardiaceae, especially within the *Schinopsis* genus, which has high concentrations of inhibitors (Mitchell 1990). Therefore, this new optimized protocol has the double advantage of circumventing DNA purification and at the same time being affordable, and thus helping to find a feasible solution to the notable difficulties to purify and amplify DNA from some Anacardiaceae (Pell 2004; Mitchell and Daly 2015).

## Methods

### Plant material

A total of 41 specimens were used. The plant material included 36 samples of *Schinopsis* spp. and five outgroups (see Additional file 1). These materials were selected covering natural populations of Argentina, Brazil, Bolivia, Paraguay and Peru.

### DNA extraction

Silica gel dried and herbarium specimen leaves were used for DNA isolation. The DNA extraction protocol was based on previous reports (Permingeat et al. 1998; Kahan 2007) with the modifications listed below. Moreover, 12 samples were extracted using the DNeasy Plant Mini Kit (Qiagen Inc., Valencia CA) as control. In order to prevent allergic reactions (dermatitis) due to the skin-irritating components, it is recommended to protect own skin in all steps of the procedure, particularly when sampling and grinding the material.

### Total DNA extraction protocol

1. Ground 20–25 mg of plant tissue in 1.5 ml Eppendorf tubes using polypropylene micropestles by hand or with the aid of a mechanic disruptor, or in a mortar and pestle with a pinch of sterile sand. (*)
2. Transfer the powder to a 1.5 ml Eppendorf tubes containing 1 ml Extraction Buffer (Tris HCl 100 mM pH 8; NaCl 1.4 M; EDTA 20 mM; glucose 0.5 M; CTAB—cetyltrimethylammonium bromide—2 %) preheated at **75** **°C**.
3. Homogenize and incubate at 75 °C for 150 min by vortering every 30 min. (*)
4. Centrifuge at 10,000 rpm for 10 min at room temperature, and transfer approximately 700 μl of the upper liquid layer to a 1.5 ml Eppendorf tube. (*)
5. Add 1 volume of chloroform and produce an emulsion by gently shaking the tube for 10 min.
6. Centrifuge at 10,000 rpm for 10 min at room temperature.
7. Repeat steps 5 and 6, but with shaking for 5 min. (*)
8. Recover 500 μl of the supernatant and transfer to a new 1.5 ml tube. Precipitate DNA with isopropanol 0.8 vol for **12** **h** at −20 °C.
9. Precipitate DNA by centrifugation at 10,000 rpm for **15** **min** at room temperature and discard the upper liquid layer.
10. Wash the precipitate with 500 μl of ethanol 75 % and centrifuge 15 min at 10,000 rpm, discarding the supernatant. (*)
11. Dry the DNA in stove at 37 °C, and then resuspend the pellet in 50 μl of TE buffer (Tris HCl 100 mM pH 8; EDTA 1 mM).
12. Add 0.5 μl of RNase A (Invitrogen) and incubate for **60** **min** at 37 °C to digest the RNA.
13. Precipitate the DNA by adding first 5 % V/V of NaCl 5 M and then 2 volumes of cold ethanol 100 % for 12 h at −20 °C. (*)
14. Centrifuge at 10,000 rpm for **15** **min** at room temperature.
15. Do a second wash with 500 μl of cold ethanol 75 % V/V, and discard the supernatant. (*)
16. Dry well the DNA in stove at 37 °C, and then dissolve in 50 μl of sterile and **distilled water**.
  (*) Steps *substantially* modified; some features are in **bold type** to indicate the parts of the steps that were *partially* modified from Permingeat et al. (1998).

After purification, the integrity of the DNA was tested by electrophoresis in 0.8–1 % agarose gel in TAE 1× buffer, at 60 mA for approximately 2.30 h. The DNA was stained with ethidium bromide (10 μg/ml) and visualized under a UV transilluminator. The DNA yield was estimated by spectrophotometric analysis and by comparing the fluorescence intensity of each sample to 100 ng of Lambda (*Eco*RI/*Hin*dIII) marker (Promega, USA) (Fig. 1a) or 325 ng of 100 bp DNA Ladder (Promega) (Fig. 1b) as standard.

### PCR amplification reactions

Both chloroplast and nuclear regions were amplified from total DNA following the PCR protocols described by Pell (2004). To amplify *trn*L-F, *rps*16 and *ndh*F regions primers in Taberlet et al. (1991), Oxelman et al. (1997) and Olmstead and Sweere (1994) were used. On the other hand, for nuclear markers, the primers reported by Weeks (2003) and Baldwin and Markos (1998) were employed for ETS regions, and the primers in White et al. (1990) and Wurdack in Pell (2004) respectively were applied to amplify ITS2 (see primers details in Additional file 2).

 PCR amplifications were carried out in 50 μl final volume reactions (Table 1) using only the DNA samples obtained with the modified protocol, diluted 1/10 in order to reduce inhibitors concentration (Savolainen et al. 1995). PCR steps, for each reaction, are summarized in Table 1. For *trn*L-F, the thermal cycle consisted on 2 min at 97 °C for one cycle, followed by 30 cycles of denaturation at 94 °C for 1 min, annealing at 48 °C for 2 min and 72 °C for 2 min and finally 16 min at 72 °C was allowed to the dissociation step. Similar parameters were used for *rps*16, except for the annealing temperature, which was 55 °C (modified from Pell 2004). For *ndh*F, we followed the parameters detailed in Davis et al. (2002) i.e.: an initial denaturation of 3 min at 94 °C, then 35 cycles of 94 °C for 30 s, 48 °C for 1 min and 72 °C for 50 s, and a final elongation of 6 min at 72 °C. Nuclear genes segments were amplified using the following parameters: 10 min at 95 °C, followed by 35 cycles of 1 min at 94 °C, 2 min at 56 °C and 2 min at 72 °C, and finally 16 min at 72 °C for ETS and 50 s at 97 °C for one cycle, then 35 cycles of 50 s at 97 °C, annealing for 50 s at 53 °C, and 1.50 min at 72 °C, and 7 min at 72 °C for ITS2 (modified from Pell 2004).

**Table 1:** List of reagents and PCR steps to amplify chloroplast regions *trn*L-F*, rps*16, *ndh*F and nuclear regions ETS and ITS2

PCR reagents	Final concentration	
	Chloroplast regions	Nuclear regions
H_2_O s&d		
Buffer 5× (Promega)	1×	1×
DMSO 1 M (Sigma-Aldrich)	–	10 %
BSA 400 ng/μl (Promega)	1/1000	1/1000
Cl_2_Mg (Promega)	5 mM	5 mM
Primer F	1 μM	1 μM
Primer R	1 μM	1 μM
dNTP’s 1 mM (Promega)	200 μM	200 μM
DNA dil 1/10	25 ng	25 ng
Taq pol 5u/μl (Promega)	1.5–2 U	1.5–2 U

PCR steps	*trn*L-F/*rps*16	*ndh*F	ETS	ITS2
1—Initialization	2 min—97 °C	3 min—94 °C	10 min—95 °C	50 s—97 °C
2—Denaturation	1 min—94 °C	30 s—94 °C	1 min—94 °C	50 s—97 °C
3—Annealing	2 min—48/55 °C	1 min—48 °C	2 min—56 °C	50 s—53 °C
4—Extension	2 min—72 °C	50 s—72 °C	2 min—72 °C	1.5 min—72 °C
5—Elongation	16 min—72 °C	6 min—72 °C	16 min—72 °C	7 min—72 °C
	[steps 2–3–4] × 30	[steps 2–3–4] × 35	[steps 2–3–4] × 35	[steps 2–3–4] × 35

For several samples, the amplification of *rps*16 produced more than one PCR product. Consequently, the target amplicons were extracted from the agarose gel and purified using Wizard SV Gel and PCR Clean-Up System (Promega, Madison, WI, USA). Then they were re-amplified following the procedure described above.

### Sequencing of PCR amplicons and bioinformatics analysis

PCR products were sequenced at Macrogen (Seoul, South Korea; http://dna.macrogen.com/eng/). Each fragment was sequenced in both directions (5′–3′ and 3′–5′) employing the same primers used in the amplification reactions (see Additional file 2). The identity of the sequences obtained was confirmed by comparison with sequences available in the National Center for Biotechnology Information (NCBI; http://www.ncbi.nlm.nih.gov/) database. For each taxon and DNA region, forward (5′–3′) and reverse (3′–5′) sequences were assembled and checked for inaccurate base pairing using the Sequencher (v. 4.1, Gene Codes Corp.) free software.

## Additional files

10.1186/s40064-016-2118-4 List of taxa and specimens used in the study.
10.1186/s40064-016-2118-4 List of PCR primers (5′-3′) to amplify chloroplast and nuclear regions.
