# Supplementary material for: Optimization of DNA extraction and PCR protocols for phylogenetic analysis in Schinopsis spp. and related Anacardiaceae
Source: Springerplus. 2016 Apr 18;5:477. doi: 10.1186/s40064-016-2118-4 (PMC4835408; doi:10.1186/s40064-016-2118-4)
Supplement: Supplementary file 1 — 10.1186/s40064-016-2118-4 List of taxa and specimens used in the study. [file 40064_2016_2118_MOESM1_ESM.docx]

Table 1 List of taxa and specimens used in the study

| **Species** | **Collector/Voucher code** | **Data col.** | **COUNTRY/State** | **Preserved/Herbarium** |
| --- | --- | --- | --- | --- |
| *S. balansae* Engl. | Vera, Mogni & Oakley 1aPY | 16/12/2011 | PARAGUAY Presidente Hayes | Silica-gel FCQ |
| *S. balansae* Engl. | Mogni, Prado, Oakley, Maturo & Albute 3aCHAC | 28/02/2011 | ARGENTINA Chaco | Silica-gel UNR |
| *S. balansae* Engl. | Prado, Oakley, Mogni & Albute 1aSESTII(SFEII) | 18/03/2011 | ARGENTINA Santiago del Estero | Silica-gel UNR |
| *S. balansae* Engl. | Cárcamo, J.M. 2aHUA | 07/12/2011 | ARGENTINA Santa Fe | Silica-gel UNR |
| *S. balansae* Engl. | Mogni, Prado, Oakley, Maturo & Albute 3aFOR | 27/02/2011 | ARGENTINA Formosa | Silica-gel UNR |
| *S. balansae* Engl. | Pott, A., Pott, V.J. & Prado 4613 | 18/02/1989 | BRAZIL Mato Grosso do Sul | Herbarium specimen at UNR |
| *S. boqueronensis* Mogni & Oakley | Vera, Mogni, & Oakley 4238 | 14/12/2011 | PARAGUAY Boquerón | Silica-gel FCQ |
| *S. boqueronensis* Mogni & Oakley | Vera, Mogni, & Oakley 4227 | 14/12/2011 | PARAGUAY Boquerón | Silica-gel FCQ |
| *S. boqueronensis* Mogni & Oakley | Vera, Mogni, & Oakley 4239 | 15/12/2011 | PARAGUAY Boquerón | Silica-gel FCQ |
| *S. boqueronensis* Mogni & Oakley | Vera, Mogni, & Oakley 4240 | 16/12/2011 | PARAGUAY Presidente Hayes | Silica-gel FCQ |
| *S. brasiliensis* Engl*.* | Daza, A. 4036 | 2005-12-03 | PERÚ Junín | Herbarium specimen at E |
| *S. brasiliensis* Engl*.* | Correia dos Santos, Mogni & Souza Lima 6fANT-BA | 07/08/2013 | BRAZIL Bahia | Silica-gel HUEFS |
| *S. brasiliensis* Engl*.* | Sao Mateus, Mogni & Bené 49fIBO-BA | 18/08/2013 | BRAZIL Bahia | Silica-gel HUEFS |
| *S. brasiliensis* Engl*.* | Sao Mateus, Mogni & Bené 50fPAR-BA | 18/08/2013 | BRAZIL Bahia | Silica-gel HUEFS |
| *S. brasiliensis* Engl*.* | Pott, A., Pott, V.J. & Prado 4611 | 17/02/1989 | BRAZIL Mato Grosso do Sul | Herbarium specimen at UNR |
| *S. brasiliensis* Engl*.* | Mogni, De Abreu Moreira & Harrison 85fAIU-CE | 12/09/2013 | BRAZIL Ceará | Silica-gel HUFRN |
| *S. cornuta* Loes. | Vera, Mogni & Oakley 1ePY | 14/12/2011 | PARAGUAY Boquerón | Silica-gel FCQ |
| *S. cornuta* Loes. | Vera, Mogni & Oakley 4ePY | 15/12/2011 | PARAGUAY Boquerón | Silica-gel FCQ |
| *S. cornuta* Loes. | Vera, Mogni & Oakley 5e?PY | 15/12/2011 | PARAGUAY Boquerón | Silica-gel FCQ |
| *S.* aff. *glabra* [(Engl.) F.A.Barkley & T.Mey.](http://www.ipni.org/ipni/idPlantNameSearch.do;jsessionid=CD47971795F163AB4811D091260ED8B6?id=229144-2&back_page=%2Fipni%2FeditSimplePlantNameSearch.do%3Bjsessionid%3DCD47971795F163AB4811D091260ED8B6%3Ffind_wholeName%3DSchinopsis%2Bglabra%26output_format%3Dnormal) | Correia dos Santos, Mogni & Souza Lima 9fGLO-BA | 08/08/2013 | BRAZIL Bahia | Silica-gel HUEFS |
| *S.* aff. *glabra* [(Engl.) F.A.Barkley & T.Mey.](http://www.ipni.org/ipni/idPlantNameSearch.do;jsessionid=CD47971795F163AB4811D091260ED8B6?id=229144-2&back_page=%2Fipni%2FeditSimplePlantNameSearch.do%3Bjsessionid%3DCD47971795F163AB4811D091260ED8B6%3Ffind_wholeName%3DSchinopsis%2Bglabra%26output_format%3Dnormal) | Correia dos Santos, Mogni & Souza Lima 20fPET-PE | 09/08/2013 | BRAZIL Pernambuco | Silica-gel HUEFS |
| *S. heterophylla* Ragonese & J.A.Castigl*.* | Prado, Oakley, Mogni & Albute 1d?CHACII | 19/03/2011 | ARGENTINA Chaco | Silica-gel UNR |
| *S. heterophylla* Ragonese & J.A.Castigl*.* | Prado, Oakley, Mogni & Albute 1bFORII | 20/03/2011 | ARGENTINA Formosa | Silica-gel UNR |
| *S. heterophylla* Ragonese & J.A.Castigl*.* | Mogni, Prado, Oakley & Chamorro 2bSESTIII | 04/07/2012 | ARGENTINA Santiago del Estero | Silica-gel UNR |
| *S. heterophylla* Ragonese & J.A.Castigl*.* | Oakley & Mogni 1bCHAR | 01/05/2010 | ARGENTINA Chaco | Silica-gel UNR |
| *S. lorentzii* (Griseb.) Engl*.* | Vera, Mogni & Oakley 1cPY | 14/12/2011 | PARAGUAY Boquerón | Silica-gel FCQ |
| *S. lorentzii* (Griseb.) Engl*.* | Prado, Oakley, Mogni & Albute 1cSESTII(SFEII) | 18/03/2011 | ARGENTINA Santiago del Estero | Silica-gel UNR |
| *S. lorentzii* (Griseb.) Engl*.* | Prado & Martínez 1cJUJII | 16/05/2011 | ARGENTINA Jujuy | Silica-gel UNR |
| *S. lorentzii* (Griseb.) Engl*.* | Prado, Maturo & Mogni 1cCATII | 21/11/2010 | ARGENTINA Catamarca | Silica-gel UNR |
| *S. lorentzii* (Griseb.) Engl*.* | Mogni, Prado, Oakley, Maturo & Albute 1cFOR | 27/02/2011 | ARGENTINA Formosa | Silica-gel UNR |
| *S. lorentzii* (Griseb.) Engl*.* | Mogni, Prado, Oakley, Maturo & Albute 1cSGUA | 27/02/2011 | ARGENTINA Santiago del Estero | Silica-gel UNR |
| *S. marginata* Engl. | Mogni, Galetti, Oakley & Prado 1dSJ | 27/05/2013 | ARGENTINA San Juan | Silica-gel UNR |
| *S. marginata* Engl. | Mogni, Galetti, Oakley & Prado 1dSALV | 28/09/2012 | ARGENTINA Salta | Silica-gel UNR |
| *S. marginata* Engl. | Toledo, C.S 2818 | 13/12/1990 | BOLIVIA, Santa Cruz | Herbarium specimen at MCNS |
| *S. peruviana* Engl. | Unknown | - | PERÚ San Martín | Herbarium specimen at E |
| *S. peruviana* Engl. | Pennington, T.D.; Pennington, R.T.; Daza, A. 17625 | 2003-12-03 | PERÚ San Martín | Herbarium specimen at E |
| *Apterokarpos gardneri* [(Engl.) Rizzini](http://www.ipni.org/ipni/idPlantNameSearch.do?id=69231-1&back_page=%2Fipni%2FeditSimplePlantNameSearch.do%3Ffind_wholeName%3DApterokarpos%2Bgardneri%26output_format%3Dnormal) | Unknown | - | BRAZIL | DNA sample from LAMOL-UEFS |
| *Astronium balansae* Engl. | Mogni, Prado, Oakley & Chamorro AST1 | 08/07/2012 | ARGENTINA Formosa | Silica-gel UNR |
| *Lithraea molleoides* (Vell.) Engl. | Mogni, Galetti, Oakley & Prado LITMO1 | 25/05/2013 | ARGENTINA Catamarca | Silica-gel UNR |
| *Loxopterygium grisebachii* [Hiern ex Griseb.](http://www.ipni.org/ipni/idPlantNameSearch.do?id=69863-1&back_page=%2Fipni%2FeditSimplePlantNameSearch.do%3Ffind_wholeName%3Dloxopterygium%2Bgrisebachii%26output_format%3Dnormal) | Prado & Martínez LOX1 | 11/02/2012 | ARGENTINA Salta | Silica-gel UNR |
| *Schinus areira* L. | Mogni SCHA1 | 22/01/2013 | ARGENTINA Santa Fe | Silica-gel UNR |
