# Supplementary material for: Optimization of DNA extraction and PCR protocols for phylogenetic analysis in Schinopsis spp. and related Anacardiaceae
Source: Springerplus. 2016 Apr 18;5:477. doi: 10.1186/s40064-016-2118-4 (PMC4835408; doi:10.1186/s40064-016-2118-4)
Supplement: Supplementary file 2 — 10.1186/s40064-016-2118-4 List of PCR primers (5′-3′) to amplify chloroplast and nuclear regions. [file 40064_2016_2118_MOESM2_ESM.docx]

Table 2 List of PCR primers (5’−3’) to amplify **A:** chloroplast regions and **B:** nuclear regions

| **A**  *trn*L-F= **e-F** GGTTCAAGTCCCTCTATCCC; **f-R** ATTTGAACTGGTGACACGAG  *rps*16= ***rps*16-F** GTGGTAGAAAGCAACGTGCGA CTT; ***rps*19-R2** TCGGGATCGAACATCAATTGCAAC  *ndh*F= **1318-F** GGATTAACYGCATTTTATATGTTTCG; **1955-R** CGATTATATGACCAATCATATA |
| --- |
| **B**  ETS= **ETS1-F** TTCGGTATCCTGTGTTGCTTAC; **18S-IGS-R** GAGACAAGCATATGACTACTGGCAGGATCAACCAG  ITS2= **ITS 3-F** GCATCGATGAAGAACGCAGC; **26S-25-R** TATGCTTAAAYTCAGCGGGT |
